# Potential of the Probiotic *Lactobacillus Plantarum* ATCC 14917 Strain to Produce Functional Fermented Pomegranate Juice

**DOI:** 10.3390/foods8010004

**Published:** 2018-12-22

**Authors:** Ioanna Mantzourani, Stavros Kazakos, Antonia Terpou, Athanasios Alexopoulos, Eugenia Bezirtzoglou, Argyro Bekatorou, Stavros Plessas

**Affiliations:** 1 Laboratory of Microbiology, Biotechnology and Hygiene, Faculty of Agriculture Development, Democritus University of Thrace, 68200 Orestiada, Greece; stkazak@yahoo.gr (S.K.); alexopo@agro.duth.gr (A.A.); empezirt@agro.duth.gr (E.B.); 2 Food Biotechnology Group, Department of Chemistry, University of Patras, GR-26504 Patras, Greece; aterpou@upatras.gr (A.T.); abekatorou@upatras.gr (A.B.)

**Keywords:** *Lactobacillus plantarum* ATCC 14917, pomegranate juice, fruit, probiotic, antioxidant activity, phenolics, functional beverage

## Abstract

In this research survey the application of probiotic strain *Lactobacillus plantarum* ATCC 14917 in pomegranate juice fermentation is sought. Pomegranate juice was fermented for 24 h and then it was stored 4 for 4 weeks. Cell viability retained in high levels after the 24 h of fermentation and storage for 4 weeks (above 8.8 log cfu/mL), while fermented pomegranate juice was scored better at the 4th week of storage compared to non-fermented pomegranate juice. The probiotic strain was effective regarding lactic acid fermentation as was proved through sugar and organic acids analysis. Concentration of ethanol was maintained at low levels (0.3–1% *v*/*v*). Fermented pomegranate juice contained more and in higher percentages desirable volatile compounds (alcohols, ketones and esters) even at the 4th week of cold storage compared to non-fermented juice. Antioxidant activity (150.63 mg Trolox equivalent (TE)/100 mL at the 2nd week) and total phenolic content (206.46 mg gallic acid equivalents (GAE)/100 mL at the 2nd week) were recorded in higher levels for all the storage time compared to non-fermented juice.

## 1. Introduction 

Functional foods are being used worldwide as agents targeting to prevent disease [1]. As a result, functional foods have been gaining significant attention from the food industry during the past few years [2,3]. The international market of functional foods is rising and represents one of the most attractive areas of innovation regarding the food sector [4,5]. In general, functional foods exert beneficial health effects and include foods that contain bioactive compounds and probiotic [1]. Probiotics are live microorganisms (mainly bacteria but also yeasts) often called “good” or “helpful” because they promote the equilibrium of intestinal microflora [6,7]. Additionally, the consumption of probiotic food is indicated by many studies to reduce the level of serum cholesterol, to enhance the immune system and to prevent colon cancer [8]. However, probiotics should be present in adequate amounts in the food matrix in order to deliver their beneficial effects to the host [7]. Likewise, the minimum concentration of viable cells for a probiotic food product has been estimated to be approximately 10^6^–10^7^ cfu/mL during the time of consumption [9]. In many studies, as well as industrial applications, the main vehicles for delivering probiotic bacteria have been demonstrated to be dairy products [10,11,12]. However, probiotic dairy products are often not suitable for consumption by certain groups of consumers due to increased incidence of lactose-intolerance, allergies, dyslipidemia and vegetarianism [13,14]. As a result of increasing consumers’ demand for alternative non-dairy substrates for the delivery of probiotic bacteria, academic and industrial research was triggered toward the development of innovative juice and vegetable probiotic beverages [15,16,17,18,19]. 

Fruit juices and beverages market is currently demonstrating a dynamic growth worldwide [20,21]. Functional beverages manufactured with fruit or vegetable juices with incorporated probiotic bacteria are considered as an attractive option for those who do not consume dairy products [19]. In addition, fruit juices have been reported as novel suitable carrier for the delivery of probiotic bacteria as they are rich in vitamins, minerals and antioxidant compounds providing a suitable growth substrate in parallel with a strong health appeal [15,16]. Fermentation of fruit juice by probiotic bacteria can increase viability of the cells and in addition improve functional aspects of the produced beverage [16,22]. As a result, a wide variety of ongoing research has recently focused on fermented juice production by the use of various probiotic strains providing outstanding results [15,22,23,24]. Among many fruits, pomegranate juice is quite appreciated for its functional properties as it has potent anti-oxidative characteristics, anti-inflammatory and antimicrobial properties and has been previously employed for fermentation by probiotic lactic acid bacteria ameliorating the health benefits of the juice [25,26,27,28].

The main aim of this study was the development of a novel fermented pomegranate beverage by the application of *Lactobacillus plantarum* ATCC 14917 [29]; a probiotic strain with good technological characteristics which was evaluated in the frame of this research. The parameters that were analyzed mainly focused on (i) concentration of residual sugars, organic acids and ethanol, (ii) volatile compounds, (iii) total phenolics content, (iv) antioxidant activity and (v) viability of the strain. To the best of our knowledge, no previous studies demonstrated the effect of pomegranate probiotic juice fermentation during cold storage (4 °C) for four weeks besides the 24 h of fermentation.

## 2. Materials and Methods

### 2.1. Microorganism

The probiotic strain *Lactobacillus plantarum* ATCC 14917 was selected and applied in the fermentations [29]. It was grown under anaerobic conditions at 37 °C for 48 h in MRS broth. Wet biomass was harvested by centrifugation (Sigma 3K12, Bioblock Scientific, Lezennes, France) at 5000 rpm for 10 min at 25 °C. All media were autoclaved at 120 °C and at 1–1.5atm for 15 min prior to use.

### 2.2. Pomegranate Juice Fermentation

Pomegranates (*Punica granatum* L.) were obtained by a local market (Orestiada, Greece). They were washed and processed into juice by blending the seeds for 10 min. Sterilized water was added to adjust the initial sugar concentration to approximately 90 g/L, and the initial pH was adjusted to 3.5 with NaOH 4N. The prepared juice solutions (100 mL) were transferred into 250 mL flasks and pasteurized for 5 min at 80 °C, cooled at room temperature and finally used for the fermentations [26]. 1 g of harvested (wet weight) *Lactobacillus plantarum* ATCC 14917 was added to 100 mL of pomegranate juice that was fermented at 30 °C for 24 h. The initial cell viability was determined at 11.42 log cfu/mL of juice. Then, the flaks were kept at 4 °C for 28 days (4 weeks). The fermentations were carried out in triplicate. 

### 2.3. Ethanol and Residual Sugar Analysis

Samples were collected at various time intervals (days 0, 1, 7, 14, 21 and 28) and were analyzed for residual sugar (glucose, fructose and sucrose), and ethanol concentration, by high performance liquid chromatography on a Shimadzu HPLC system (Shimadzu, Kyoto, Japan) consisting of a SCR-101N stainless steel column, a LC-9A pump, a CTO-10A oven set at 60 °C and a RID-6A refractive index detector. Ultra-pure water obtained by a Milli-Q water purifying system (resistivity 18.2 MΩ cm^−1^, Darmstadt, Germany).) was used as mobile phase with a flow rate of 0.8 mL/min, and 1-butanol (0.1% *v*/*v*) was used as internal standard. Samples were filtered through 0.2 µm microfilters, before injection. Ethanol (% *v*/*v*) and residual sugar (g/L) concentrations were calculated using standard curves. All results are presented as means of at least three repetitions plus standard deviations.

### 2.4. Organic Acid Analysis

Organic acids (lactic and acetic) were determined by ion-exchange liquid chromatography as described before by Plessas, et al. [30]. The analysis was performed on an ion-exchange HPLC Shimadzu system consisting of a Shim-pack ICA1 column, an LC-10AD pump, a CTO-10A oven, and a CDD-6A conductivity detector. A solution of 2.5 mM phthalic acid and 2.4 mM tris (hydroxymethyl) aminomethane (pH 4.0) was used as mobile phase (1.2 mL/min). The column temperature was 40 °C. The sample dilution was 5% *v*/*v*, and the injection volume was 60 µL. Determinations were carried out using standard curves.

### 2.5. Microbiological Analysis

Aliquots of 10 mL were collected from each pomegranate juice (after homogenisation by shaking thoroughly) at various time intervals during fermentation and storage. The samples were blended with 90 mL of sterile 1/4 strength Ringer’s solution (Sigma-Aldrich) and mixed in a stomacher blender and subjected to serial decimal dilutions in 1/4 strength Ringer’s solution. Viable counts of lactobacilli, yeasts and fungi, and coliforms were determined in triplicate by plating appropriate dilutions on the selective media for each species [31]. Specifically, viable counts of *Lactobacillus plantarum* were enumerated on acidified MRS agar (Merck, Darmstadt, Germany) at 37 °C for 72 h, anaerobically (Anaerobic jar, Anerocult C, Merck, Darmstadt, Germany). Coliforms were enumerated on Violet Red Bile agar (Lab M, Lancashire, UK) after incubation at 30 °C for 24 h. Yeasts and fungi were determined by plating on Sabouraud Chloramphenicol Agar (Merck, Germany) after incubation at 30 °C for 72 h. All cell counts were expressed as log of mean colony forming units (cfu) per mL of pomegranate juice. All results are presented as means of three repetitions plus standard deviations. 

### 2.6. Total Phenolics and Antioxidant Activity

Total phenolic content was determined by using the Folin-Ciocalteu reagent (Sigma, St. Louis, MO, USA) based on colorimetric reduction [32]. The phenolic compounds are oxidized to phenolates by the reagent at alkaline pH in a saturated solution of sodium carbonate resulting in a blue complex. About 1mL of Folin–Ciocalteau (10%, *w*/*v*,) is added to 0.2 mL of prepared pomegranate juice, followed by the addition of 1.2 mL of aqueous Na_2_CO_3_ (7.5%, *w*/*v*). The mixture was left in the dark for 90 min. The absorbance of the blue color solution was monitored at 760 nm on a UV visible spectrophotometer (Shimadzu, Kyoto, Japan), against blank (distilled water). The total phenolics content (TPC) was assessed by plotting the gallic acid calibration curve and expressed as mg of gallic acid equivalents (GAE)/100 ml juice. The antioxidant activity (AA) of pomegranate juices was evaluated applying the ABTS radical cation decolorization assay [33]. ABTS^+^ was prepared by reacting of ABTS with potassium persulfate. Samples were analyzed at five different dilutions, within the linearity range of the assay, as previously described by Gentile, et al. [34]. TAA was expressed as mg of Trolox equivalent (TE)/100 mL juice. All measurements were repeated three times. All measurements were repeated three times.

### 2.7. Volatiles Analysis by HS-SPME/GC-MS

The volatiles of the fermented pomegranate juices were determined using Gas Chromatography/Mass Spectrometry with Headspace Solid-Phase Micro-Extraction sampling (HS-SPME/GC-MS), as described by Vázquez-Araújo, et al. [35] with small modifications. Each sample (2 mL) was pipetted into 4 mL glass vial and sealed with a screw-cap with PTFE-lined silicone septum. The vials were placed in a water-bath at 40 °C and magnetically stirred at 250 rpm for 5 min before exposing the fibre (DVB/CAR/PDMS, needle size 24 ga, length 1 cm, Sigma Aldrich) for 30 min at the same conditions. Desorption of volatiles was affected at 250 °C for 2 min (splitless) in the inlet of GC-MS system (Shimadzu QP-2010 Ultra). The fibre was then held in the inlet (split ratio 1/50) for another 8 min to prevent carryover effects. Compounds were separated on a MEGA-5 HT column (30 m × 0.25 mm i.d., film thickness 0.25 μm, Mega s.n.c., Legnano, Italy) using helium as a carrier gas at a constant linear velocity (35 cm/s). During analysis, the oven was kept at 40 °C for 5 min, then increased with 4 °C/min up to 150 °C followed by 30 °C/min up to 260 °C, and held for 5 min. The mass spectrometer was operated in the electron ionization mode with the electron energy set at 70 eV and scan mass range of 40–400 m/z. Source and interface temperatures were set at 200 and 270 °C respectively. Identification of the compounds was affected by comparing: (i) the linear retention indices based on the homologous series of n-alkanes (C7-C24) with those of reference compounds and those of NIST14 and FFNSC MS library (Chromaleont S.r.l., Messina, Italy), (ii) MS data with those of reference compounds and by MS data obtained from NIST14 and FFNSC libraries. GC-MS solution (Shimadzu) and Amdis (National Institute of Standards and Technology—NIST) software were used in the identification process. The relative amounts of individual components were calculated on the basis of peak area (from Amdis) without using any correction factor.

### 2.8. Sensory Evaluation

Sensory evaluation of the fermented pomegranate beverages was performed by a panel of 30 non-trained laboratory members who scored the aroma, taste and overall acceptability in comparison with commercial pomegranate juice, after the end of juice fermentation and during storage at 4 °C [36]. The samples were coded by a different 3-digital number and were served in a randomized order, while the panel was asked to evaluate them based on 0–10 preference scale. The results are presented as average scores plus standard deviations

### 2.9. Statistical Analysis

The data obtained from physicochemical characteristics, antioxidant activity, total phenolics content and cell viability of the non-fermented and fermented pomegranate juice were analyzed for their mean differences with the Analysis of Variance (ANOVA) procedure followed by Duncan’s post hoc multiple range test to extract the specific differences between the various treatments. Analysis was performed by using IMB SPSS v20 (IBM Corp., Armonk, NY, USA) at an alpha level of 5%.

## 3. Results and Discussion

### 3.1. Cell Viability

The viability of *Lactobacillus plantarum* ATCC 14917 as well as possible spoilage by yeasts and fungi or coliforms were recorded after juice fermentation and during the four weeks of storage at 4 °C (Table 1). According to the results, cell viability of the probiotic *Lactobacillus plantarum* strain was maintained at high levels throughout three weeks of cold storage (above 10 log cfu/mL), while decreased during the last week of storage (statistically significant). Specifically, viable probiotic cell counts were decreased to 8.83 log cfu/mL at the last week of storage (4th). However, even in this case, the recorded viability value was above the limit of 6–7 log cfu/mL, which is required for probiotic products [9]. At this point, it should be underlined that, the initial pH value of the freshly prepared pomegranate juice used in this study was approximately 3.0. Likewise, before fermentation, a slight increase of the substrate pH (with NaOH 4N) was made to a value of 3.5, so as to make the pomegranate juice more fermentable by *Lactobacillus plantarum* ATCC 14917 (Table 1). 

A possible explanation of the high levels of *Lactobacillus plantarum* ATCC 14917 viability during storage is that lactic acid fermentation might have increased the bio accessibility of phenolic compounds. There are reports from the literature claiming that phenolic compounds may act as prebiotics [37]. Likewise, possible prebiotic activity led to the amelioration of the growth of *Lactobacillus plantarum* ATCC 14917. In addition, it has been noted that some strains of *Lactobacillus plantarum* can grow in fruit matrices due to their tolerance to acidic environments [38]. Indeed, *Lactobacillus plantarum* ATCC 14917 has been reported to exhibit high acid resistance ability [29]. Furthermore, no spoilage of the fermented pomegranate juice, by yeasts, fungi and coliforms was observed even after the 4th week of storage at 4 °C (Table 1). It seems that lactic acid fermentation of pomegranate juice could provide a protective effect from microbiological spoilage as also reported by previous studies [39,40].

### 3.2. Ethanol, Organic Acids and Residual Sugar Concentrations

The results obtained for residual sugar, lactic and acetic acid and ethanol are presented in Table 2.

According to the results, residual sugar levels were decreased while the levels of organic acids were increased, demonstrating the efficiency of the strain *Lactobacillus plantarum* ATCC 14917 for lactic acid fermentation of pomegranate juice. In particular, the residual sugars concentration was reduced (statistically significant) by approximately 20% (65.0 g/L) and 23% (62.9 g/L) after the 3rd and the 4th weeks of storage respectively. On the other hand, the level of lactic acid increased (statistically significant) every week reaching its maximum value at the 4th week of storage (3.75 g/L), while acetic acid concentration was determined after the 2nd week reaching its maximum value at the last week (0.86 g/L). Ethanol concentration significantly increased from 0.3% after 24 h of fermentation to 1.0% (*v*/*v*) at the end of the 3th and 4th week of storage. 

### 3.3. Total Phenolics and Antioxidant Activity

**Figure 1:**
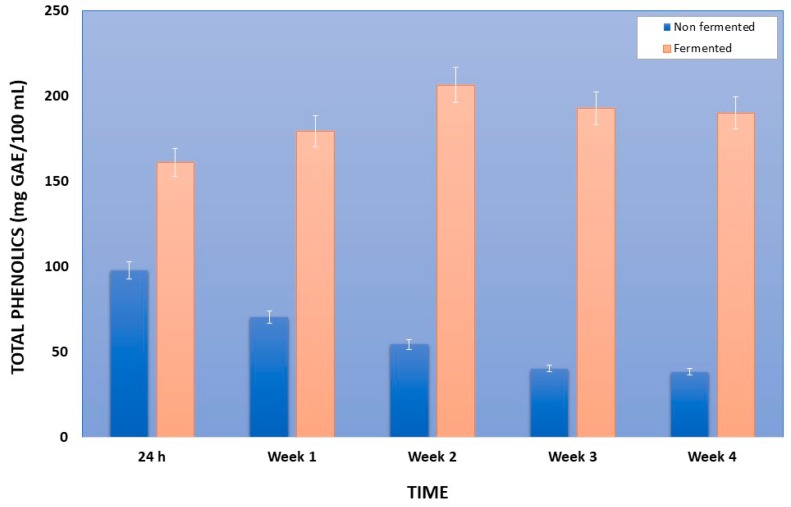
Total phenolics of fermented with *Lactobacillus plantarum* ATCC 14917 and non-fermented pomegranate juice and at first 24 h at 30 °C and during storage at 4 °C for 4 weeks. GAE, gallic acid equivalents.

The results concerning the total phenolics content (TPC) and antioxidant activity (AA) of non-fermented and fermented pomegranate juice by *Lactobacillus plantarum* ATCC 14917 are presented in Figure 1. 

Initial total phenolics content of freshly prepared pomegranate juice was about 111 ± 10 mg GAE/100 mL. The total phenolics content (TPC) of the fermented pomegranate juice significantly increased during the 24 h fermentation and was higher in all 4 weeks of storage compared to the respective values of non-fermented pomegranate juice. Specifically, after the first 24-h fermentation, total phenolics content of the fermented pomegranate juice significantly increased to an average of 161.04 mg GAE/100 mL, compared to the respective value of the non-fermented juice (control) that was decreased to 97.94 mg GAE/100 mL, (Figure 1). This statistically significant increase of TPC of fermented pomegranate juice was observed also in all the weeks of storage time (4 weeks), reaching its maximum value at the 2nd week (206.46 mg GAE/100 mL), while TPC of non-fermented pomegranate juice decreased to 38.43 mg GAE/100 mL at the 4th week of storage. It has been reported in the literature that lactic acid fermentation enhances the total phenolics content of fruit juices including pomegranate [41,42,43]. Other researchers who have demonstrated the same outcome reported that improvements in TPC of pomegranate juice can be related to the increase in the free form of phenolic compounds through the fermentation and the production of new phenolic derivatives such as catechin and α-punicalagin [37,44,45].

**Figure 2:**
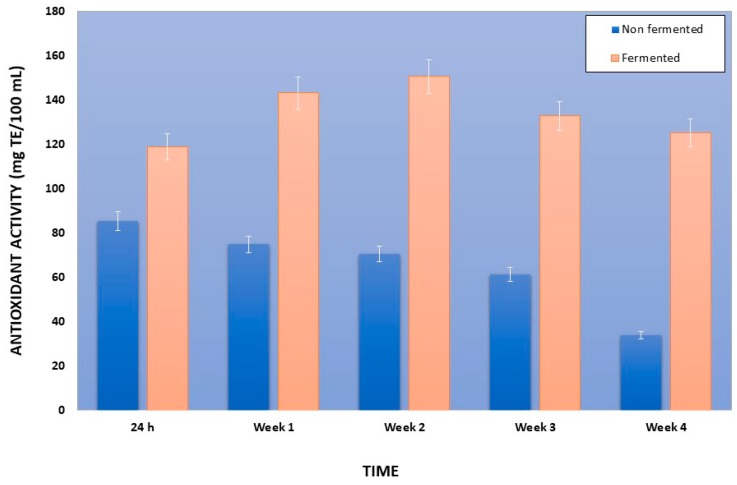
Antioxidant activity of fermented with *Lactobacillus plantarum* ATCC 14917 and non-fermented pomegranate juice and at first 24 h at 30 °C and during storage at 4 °C for 4 weeks. TE, Trolox equivalent.

Regarding the antioxidant activity (AA) of fermented pomegranate juice similar outcome was observed as in the case of TPC (Figure 2). Particularly, initial AA of freshly prepared pomegranate juice was about 90 ± 15 mg TE/100 mL. During the first 24-h fermentation AA of the fermented pomegranate juice significantly increased to an average of 119.05 mg TE/100 mL, compared to the respective value of the non-fermented juice (control) that was decreased to 85.33 mg TE/100 mL (Figure 2). This statistically significant increase of AA of fermented pomegranate juice was observed also in all the weeks of storage time (4 weeks), showing an increase of approximately 32% the 3rd week of storage (132.79 mg TE/100 mL), while AA of non-fermented pomegranate juice decreased constantly (approximately 60%) to 33.98 mg TE/100 mL the last week of storage. A possible explanation of this finding is that lactic acid fermentation ameliorated the AA of pomegranate juice which is in accordance with other reports in literature [46]. Specifically, other researchers have demonstrated that some bacteria are capable of producing β-galactosidase catalyzing the release of phenolic compounds from the bonded sugar [47]. This process may lead to an increase in the antioxidant activity after fermentation [48,49,50].

### 3.4. Volatiles Composition and Sensory Evaluation

The composition of headspace volatile compounds that were identified by SPME GC/MS in the non-fermented (NF) and fermented pomegranate (F) juice at 0 and 24 h of fermentation as well as at the 4th week of storage is presented in Table 3. Statistical analysis was conducted between normalized peak area % of each volatile of NF and F pomegranate juice. Alcohols, aldehydes, ketones and esters were the predominant compounds identified. In particular, 13 alcohols, 11 aldehydes, 11 ketones, 10 esters, 7 terpenoids and furfural were identified in the fermented pomegranate juice. All these compounds have been previously identified in pomegranate juices/fruit/seeds, fermented pomegranate juice by lactic acid bacteria and other fruits (apples, berries, plums, citrus, exotic fruit, etc.) or fruit beverages [35,51,52,53,54,55,56,57].

Fermented pomegranate juice (F) seems to contain more desirable compounds compared to non-fermented pomegranate juice (NF). Specifically, F contained more and higher amounts of alcohols, ketones and esters and less amounts of aldehydes after 24 h of fermentation and at the 4th week of storage. This outcome is very significant since it is well established in the literature that: (i) alcohols contribute positively in the flavor profile due to floral attributes such as 2-ethyl-1-hexanol [51], (ii) aldehydes are non-desirable compounds in pomegranate juices [51,58], (iii) ketones can deliver several positive sensory/aroma attributes [59] and (iv) esters play an important role in contributing to fruity notes of fruit juices [60]. Likewise, it is obvious that lactic acid fermentation affected positively the flavor of pomegranate juice. Finally, various common terpenes originating from pomegranate juice [35,56] were found in both non-fermented and fermented pomegranate juice. In particular, p-cymene, d-limonene, eucalyptol, linalool, camphor, terinen-4-ol and α-terpineol were identified.

The results regarding the preliminary sensory evaluation performed by non-trained testers (consumers) for the evaluation of the produced fermented or non-fermented juices in terms of aroma, taste and overall quality (preference) are presented in Table 4. No statistically significant differences were observed except from the 4th week of storage. In that time the consumers preferred more (statistically significant) the fermented pomegranate juice in terms of aroma, taste and overall quality compared to the non-fermented pomegranate juice. 

This interesting finding can be credited to the lactic acid fermentation because it has been stated that lactic acid fermentation can enhance the flavor profile of pomegranate juice and ensure a better control of flavor changes during juice processing [51].

## 4. Conclusions

Application of probiotic *Lactobacillus plantarum* ATCC 14917 in fermentation of pomegranate juice led to the production of a functional fruit beverage with low alcoholic degree. In particular, fermented pomegranate juice had higher levels of total phenolics content and antioxidant activity compared to non-fermented pomegranate juice during all the period of storage studied. In addition, *Lactobacillus plantarum* ATCC 14917 retained its viability in high levels (above 8.8 log cfu/mL). Concerning sensorial tests conducted, no significant differences were found between fermented and non-fermented pomegranate juice except from the last week where consumers preferred the fermented juice. Therefore, the tested stain may be used for the production of a novel functional food with high concentration of probiotic bacteria, high nutritional value and acceptance from the consumers. 

## Figures and Tables

**Table 1:** Viability of the *Lactobacillus plantarum* ATCC 14917 cells in the fermented pomegranate juices after fermentation (24 h in 30 °C) and over 4 weeks of storage at 4 °C.

Temperature (°C)	Time	Viability (log cfu/mL)		
		*Lactobacillus Plantarum* ATCC 14917	Yeasts & Fungi	Coliforms
30	0	11.42 ± 0.16 ^a^	0	0
30	24 h	10.51 ± 0.15 ^a^	0	0
4	Week 1	10.23 ± 0.94 ^a^	0	0
4	Week 2	10.54 ± 0.26 ^a^	0	0
4	Week 3	10.28 ± 0.27 ^a^	0	0
4	Week 4	8.83 ± 0.58 ^b^	0	0

Similar superscript letters in columns denote no significant differences at an alpha = 0.05 (ANOVA, Duncan Post Hoc Multiple Comparisons).

**Table 2:** Analysis of sugars, organic acids and ethanol in the pomegranate juices fermented by *L. plantarum* ATCC 14917 at first 24 h at 30 °C and during storage at 4 °C for 4 weeks.

Time	Sugars (g/L)	Lactic Acid (g/L)	Acetic Acid (g/L)	Ethanol (% *v*/*v*)
24 h	82.6 ± 0.5 ^a^	1.26 ± 0.07 ^a^	<0.1	0.3 ± 0.1 ^a^
Week 1	75.3 ± 0.9 ^b^	2.86 ± 0.08 ^b^	<0.1	0.43 ± 0.06 ^a^
Week 2	75.2 ± 2.1 ^b^	2.87 ± 0.06 ^b^	0.30 ± 0.10 ^a^	0.73 ± 0.06 ^b^
Week 3	65.0 ± 0.8 ^c^	3.12 ± 0.07 ^c^	0.43 ± 0.05 ^b^	1.0 ± 0.1 ^c^
Week 4	62.9 ± 0.8 ^c^	3.75 ± 0.09 ^d^	0.86 ± 0.05 ^c^	1.0 ± 0.1 ^c^

Similar superscript letters in columns denote no significant differences at an alpha=0.05 (ANOVA, Duncan Post Hoc Multiple Comparisons).

**Table 3:** Volatile compounds identified in the non-fermented (NF) and fermented pomegranate juice (F) by Lactobacillus plantarum ATCC 14917 at 0 h and 24 h of fermentation as well at the 4th week of storage.

Compound	RI ^1^	Normalized Peak Area %					Identification ^2^
		0 h	24 h		4th week		
			F	NF	F	NF	
**Alcohols**							
Ethyl alcohol	467	0.4 ± 0.1	10.0 ± 0.8 ^a^	6.0 ± 0.1 ^b^	44.6 ± 1.2 ^a^	19.8 ± 0.8 ^b^	MS, RI, ref
1-Butanol	633	3.4 ± 0.1	6.1 ± 0.2 ^a^	2.0 ± 0.3 ^b^	2.5 ± 0.7 ^b^	10.8 ± 0.1 ^a^	MS, RI, ref
3-Methyl-1-butanol	726	2.2 ± 0.4	3.0 ± 0.3 ^a^	1.0 ± 0.3 ^b^	3.8 ± 0.6	nd	MS, RI, ref
2-Methyl-1-butanol	728	0.9 ± 0.1	<0.1	0.6 ± 0.1 ^a^	1.0 ± 0.1 ^b^	1.7 ± 0.1 ^a^	MS, RI, ref
3-Methyl-3-buten-1-ol	724	1.2 ± 0.1	7.0 ± 0.5 ^a^	2.0 ± 0.3 ^b^	0.7 ± 0.1	nd	MS, RI, ref
(E)-3-Hexen-1-ol	854	3.9 ± 0.1	3.4 ± 0.3	nd	5.4 ± 0.2	nd	MS, RI, ref
(Z)-3-Hexen-1-ol	864	<0.1	0.8 ± 0.2 ^b^	6.5 ± 0.3 ^a^	1.6 ± 0.2	nd	MS, RI, ref
1-Hexanol	869	15 ± 1.2	4.8 ± 0.2 ^a^	1.5 ± 0.2 ^b^	6.1 ± 0.3	nd	MS, RI, ref
2-Heptanol	903	0.4 ± 0.1	<0.1	1.8 ± 0.1 ^a^	nd	nd	MS, RI, ref
2-Ethyl-1-hexanol	1032	0.9 ± 0.2	1.3 ± 0.1	nd	2.4 ± 0.2	nd	MS, RI, ref
1-Nonanol	1177	1.5 ± 0.2	1.0 ± 0.1	nd	nd	nd	MS, RI, ref
1-Decanol	1413	0.3 ± 0.1	0.5 ± 0.1 ^ab^	0.5 ± 0.1 ^ab^	nd	nd	MS, RI, ref
1-Dodecanol	1480	0.3 ± 0.1	0.5 ± 0.1 ^ab^	0.5 ± 0.1 ^ab^	nd	nd	MS, RI, ref
**Aldehydes**							
Acetaldehyde	459	0.4 ± 0.1	0.8 ± 0.1	<0.1	<0.1	nd	MS, RI
3-Methyl-butanal	615	1.0 ± 0.1	0.6 ± 0.2 ^b^	2.8 ± 0.2 ^a^	<0.1	6.1 ± 0.3	MS, RI
2-Methyl-butanal	630	0.7 ± 0.1	0.5 ± 0.1 ^b^	3.9 ± 0.1 ^a^	<0.1	5.1 ± 0.2	MS, RI
Hexanal	795	1.1 ± 0.3	0.6 ± 0.1 ^a^	0.3 ± 0.1 ^b^	<0.1	2.9 ± 0.1	MS, RI, ref
Heptanal	903	0.4 ± 0.1	<0.1	6.5 ± 0.8	<0.1	11.2 ± 0.1	MS, RI
Benzaldehyde	957	<0.1	<0.1	13.2 ± 1.1	<0.1	1.2 ± 0.1	MS, RI, ref
Octanal	1004	<0.1	<0.1	2.7 ± 0.4	<0.1	7.5 ± 0.8	MS, RI, ref
Benzeneacetaldehyde	1042	<0.1	1.3 ± 0.1 ^b^	12.4 ± 0.8 ^a^	0.9 ± 0.1 ^b^	11.5 ± 0.9 ^a^	MS, RI
Nonanal	1105	0.5 ± 0.2	11.7 ± 0.9 ^a^	9.0 ± 0.4 ^b^	2.0 ± 0.1 ^b^	8.0 ± 0.4 ^a^	MS, RI
Undecanal	1310	<0.1	<0.1	2.4 ± 0.4 ^a^	nd	4.5 ± 0.5	MS, RI
Dodecanal	1412	<0.1	<0.1	5.0 ± 0.3 ^a^	nd	2.1 ± 0.2	MS, RI
**Ketones**							
2,3-Butanedione	533	0.7 ± 0.1	<0.1	<0.1	<0.1	nd	MS, RI, ref
2-Butanone	542	1.9 ± 0.3	0.7 ± 0.1 ^b^	1.5 ± 0.2 ^a^	1.1 ± 0.1	nd	MS, RI
2-Pentanone	678	2.9 ± 0.3	0.9 ± 0.1 ^a^	0.5 ± 0.1 ^b^	0.9 ± 0.1	nd	MS, RI
3-Pentanone	700	2.5 ± 0.1	1.9 ± 0.1 ^a^	<0.1	8.1 ± 0.1	nd	MS, RI
3-Hexanone	777	1.1 ± 0.2	1.6 ± 0.1 ^a^	0.8 ± 0.1 ^b^	0.8 ± 0.1	nd	MS, RI
2-Hexanone	784	1.1 ± 0.1	2.8 ± 0.3 ^a^	1.6 ± 0.1 ^b^	1.1 ± 0.1	nd	MS, RI
2-Heptanone	893	0.4 ± 0.1	1.6 ± 0.1 ^ab^	0.6 ± 0.1 ^ab^	2.5 ± 0.1	<0.1	MS, RI
3-Heptanone	887	0.3 ± 0.1	2.3 ± 0.1 ^ab^	0.6 ± 0.1 ^a^	2.5 ± 0.1	<0.1	MS, RI
4-Methyl-2-heptanone	939	14.5 ± 1.4	9.1 ± 0.3 ^a^	1.2 ± 0.1 ^a^	8.4 ± 0.4	nd	MS, RI
6-Methyl-5-hepten-2-one	990	0.4 ± 0.1	<0.1	0.8 ± 0.1 ^a^	nd	0.6 ± 0.1	MS, RI
2-Nonanone	1094	<0.1	1.1 ± 0.1 ^b^	0.5 ± 0.1 ^a^	nd	0.8 ± 0.1	MS, RI
**Esters**							
Methyl acetate	494	1.9 ± 0.2	1.5 ± 0.1 ^ab^	1.3 ± 0.1 ^ab^	0.8 ± 0.1 ^ab^	0.4 ± 0.1 ^ab^	MS, RI, ref
Ethyl acetate	560	1.9 ± 0.1	1.4 ± 0.2 ^a^	1.8 ± 0.1 ^b^	1.0 ± 0.2 ^a^	1.5 ± 0.1 ^b^	MS, RI, ref
n-Propyl acetate	711	0.6 ± 0.1	0.9 ± 0.1	nd	0.9 ± 0.1	nd	MS, RI, ref
Ethyl propanoate	709	0.4 ± 0.1	1.0 ± 0.1	<0.1	0.9 ± 0.1	nd	MS, RI, ref
Isobutyl acetate	765	1..0 ± 0.3	1.3 ± 0.1 ^a^	0.6 ± 0.1 ^b^	1.2 ± 0.1	nd	MS, RI, ref
2-Methyl-2-butyl acetate	805	2.0 ± 0.3	1.4 ± 0.8 ^a^	0.5 ± 0.1 ^b^	1.3 ± 0.1	nd	MS, RI, ref
3-Methyl-1-butyl acetate	877	0.8 ± 0.2	1.3 ± 0.1 ^b^	0.9 ± 0.1 ^a^	nd	nd	MS, RI, ref
Methyl benzoate	1093	0.8 ± 0.2	1.2 ± 0.3	nd	1.4 ± 0.7	nd	MS, RI
Ethyl octanoate	1201	<0.1	1.2 ± 0.1	nd	nd	nd	MS, RI
Ethyl decanoate	1400	0.6 ± 0.1	0.9 ± 0.1^a^	nd	<0.1	nd	MS, RI
**Terpenoids**							
p-Cymene	1021	1.4 ± 0.1	2.2 ± 0.3	<0.1	1.0 ± 0.1 ^a^	0.7 ± 0.1 ^b^	MS, RI, ref
d-Limonene	1025	0.6 ± 0.1	2.4 ± 0.7 ^a^	0.3 ± 0.1 ^b^	1.8 ± 0.1 ^a^	0.9 ± 0.1 ^b^	MS, RI, ref
Eucalyptol	1027	0.6 ± 0.1	1.1 ± 0.1	<0.1	1.4 ± 0.3 ^a^	0.9 ± 0.1 ^b^	MS, RI, ref
Linalool	1100	0.4 ± 0.1	1.6 ± 0.2 ^a^	0.4 ± 0.1 ^b^	<0.1	<0.1	MS, RI, ref
Camphor	1139	0.5 ± 0.1	1.5 ± 0.1 ^ab^	0.8 ± 0.1 ^ab^	0.8 ± 0.1 ^a^	0.3 ± 0.1 ^b^	MS, RI, ref
Terpinen-4-ol	1174	0.4 ± 0.1	1.0 ± 0.1 ^a^	0.7 ± 0.1 ^b^	<0.1	0.6 ± 0.1	MS, RI, ref
a-Terpineol	1189	2.3 ± 0.1	2.0 ± 0.1 ^a^	1.5 ± 0.1 ^b^	1.1 ± 0.1	<0.1	MS, RI
**Others**							
Furfural	705	12.6 ± 0.1	0.2 ± 0.1 ^b^	2.0 ± 0.1 ^a^	<0.1	0.9 ± 0.1	MS, RI, ref

^1^ RI = Experimental retention indices based on the homologous series of n-alkanes (C7-C24); ^2^ MS = Identification confirmed by MS, mass spectra; RI = retention indices provided with NIST14 and FFNSC mass spectral library; ref = identified by comparison to authentic compounds. Unless confirmed by comparison to authentic standards, compounds are considered as tentatively identified.; ^a–b^ Different superscript letters in a row at the same time period for non-fermented and fermented pomegranate juice indicates statistically significant differences (ANOVA, Duncan’s multiple range test, *p* < 0.05); nd = not detected.

**Table 4:** Preliminary sensory evaluation of non-fermented and fermented pomegranate juice with *Lactobacillus plantarum* ATCC 14917 cells during 4 weeks of cold storage.

Storage Time	Substrate	Aroma	Taste	Overall Quality
24 h	Non-fermented	8.6 ± 0.1 ^a^	8.5 ± 0.1 ^a^	8.1 ± 0.1 ^a^
Fermented	8.6 ± 0.1 ^a^	8.5 ± 0.1 ^a^	8.2 ± 0.1 ^a^
Week 1	Non-fermented	7.7 ± 0.1 ^b^	7.6 ± 0.1 ^b^	7.8 ± 0.1 ^b^
	Fermented	7.6 ± 0.1 ^b^	7.7 ± 0.06 ^b^	7.8 ± 0.1 ^b^
Week 2	Non-fermented	7.2 ± 0.1 ^c^	7.1 ± 0.06 ^c^	7.2 ± 0.1 ^c^
	Fermented	7.3 ± 0.1 ^b^	7.2 ± 0.1 ^c^	7.2 ± 0.1 ^c^
Week 3	Non-fermented	6.7 ± 0.1 ^c^	6.4 ± 0.1 ^d^	6.4 ± 0.1 ^d^
	Fermented	6.9 ± 0.1 ^c^	6.5 ± 0.1 ^d^	6.4 ± 0.1 ^d^
Week 4	Non-fermented	5.6 ± 0.1 ^f^	5.3 ± 0.15 ^f^	5.2 ± 0.1 ^f^
	Fermented	6.2 ± 0.1 ^d^	6.2 ± 0.1 ^d^	6.2 ± 0.1 ^d^

Similar superscript letters in columns denotes no significant differences at an alpha = 0.05 (ANOVA, Duncan Post Hoc Multiple Comparisons).
